# Biochemical characterisation of fumarase C from a unicellular cyanobacterium demonstrating its substrate affinity, altered by an amino acid substitution

**DOI:** 10.1038/s41598-019-47025-7

**Published:** 2019-07-23

**Authors:** Noriaki Katayama, Masahiro Takeya, Takashi Osanai

**Affiliations:** School of Agriculture, Meiji University, 1-1-1, Higashimita, Tama-ku, Kawasaki, Kanagawa 214-8571 Japan

**Keywords:** Bacteria, Enzymes

## Abstract

The tricarboxylic acid cycle produces NADH for oxidative phosphorylation and fumarase [EC 4.2.1.2] is a critical enzyme in this cycle, catalysing the reversible conversion of fumarate and l-malate. Fumarase is applied to industrial l-malate production as a biocatalyst. l-malate is used in a wide range of industries such as food and beverage, pharmacy chemistry. Although the biochemical properties of fumarases have been studied in many organisms, they have not been investigated in cyanobacteria. In this study, the optimum pH and temperature of *Synechocystis* 6803 fumarase C (*Sy*FumC) were 7.5 and 30 °C, respectively. The *K*_m_ of *Sy*FumC for l-malate was higher than for fumarate. Furthermore, *Sy*FumC activity was strongly inhibited by citrate and succinate, consistent with fumarases in other organisms. Substitution of alanine by glutamate at position 314 of *Sy*FumC changed the *k*_cat_ for fumarate and l-malate. In addition, the inhibitory effects of citrate and succinate on *Sy*FumC activity were alleviated. Phylogenetic analysis revealed cyanobacterial fumarase clades divided in non-nitrogen-fixing cyanobacteria and nitrogen-fixing cyanobacteria. *Sy*FumC was thus biochemically characterised, including identification of an amino acid residue important for substrate affinity and enzymatic activity.

## Introduction

Fumarase, or fumarate hydratase [EC 4.2.1.2], is an enzyme in the tricarboxylic acid (TCA) cycle and is conserved in all organisms. Fumarase catalyses the reversible conversion of fumarate and l-malate. Two classes of fumarases are found in prokaryotes, namely Class I fumarases that are iron-dependent proteins, and Class II fumarases which resemble eukaryotic enzymes belonging to the aspartase/fumarase superfamily^1^. *Escherichia coli* possesses three genes encoding fumarases, *fumA*, *fumB*, and *fumC*, which are differently regulated by oxygen and growth conditions^2,3^. In *E. coli*, FumA and FumB are Class I enzymes and FumC is a Class II enzyme and no homology exists between the two classes of fumarases^4^. The FumC protein of *E. coli* resembles fumarases found in other bacteria such as *Bacillus subtilis* as well as in mammals^5^; it is an iron-independent enzyme and relatively heat-stable compared to FumA, and its biochemical properties are similar to those of mammalian fumarases^6^. The crystal structures of *E. coli* and *Saccharomyces cerevisiae* FumC have been resolved and their activity and regulatory domains determined^7,8^. Fumarase is thus a well-studied enzyme; however, recent studies have demonstrated that human fumarases are localized not only in mitochondria but also in the cytosol and function in response to DNA damage^9,10^. A Class II fumarase in *B. subtilis* also functions in response to DNA damage by producing l-malate, which regulates translation of RecN^11^. For the application, malate is used mainly in the food and beverage industry as an acidulant, flavor enhancer, food additive and a precursor for pharmaceutical chemicals^12^. Improvement of the thermostability of *Corynebacterium glutamicum* fumarase enhances l-malate production^13^ and an enzyme membrane reactor with immobilized fumarase is used to produce l-malate^14^. For the metabolic engineering, the production of l-malate is increased by expressing yeast fumarase in *Aspergillus oryzae*, suggesting that fumarase is a rate-limiting enzyme of malate production^15^. In this way, the importance of fumarases is conserved across the kingdoms and recognized in both basic and applied sciences.

Cyanobacteria are photosynthetic prokaryotes and are known as a model for photosynthetic organisms. Cyanobacteria fix carbon dioxide and this activity influences the global carbon cycle, with cyanobacteria contributing nearly 30% of the global net primary production^16^. Moreover, engineered cyanobacteria can produce industrially relevant chemicals from fixed carbon dioxide and are suitable for biofuel and bulk chemical production^17,18^. Among cyanobacteria, *Synechocystis* sp. PCC 6803 (hereafter referred to as *Synechocystis* 6803) is a unicellular, non-nitrogen-fixing cyanobacterium, the genome of which was first sequenced in 1996^19^. Substrains of this species are used for studies of photosynthesis and for metabolic engineering^20–22^. Transcriptomic analyses using microarrays can elucidate the mechanisms of environmental stresses and identify regulatory factors that transduce environmental signals^23–25^ and transcriptional regulators of *Synechocystis* 6803 carbon metabolism have been identified using these techniques^26–28^. Subsequently, metabolomic analyses of *Synechocystis* 6803 was performed and the carbon distribution and flux in response to environmental changes and genetic manipulation were demonstrated^29–32^. Fluxome analyses showed that TCA cycle and of acetyl-CoA metabolite pool sizes are smaller in *Synechocystis* 6803 than in *E. coli*^30,32^. Furthermore, fluxes in the TCA cycle remain low under all conditions tested^30,33^, indicating that the TCA cycle of unicellular cyanobacteria is unique among bacteria.

The cyanobacterial TCA cycle was thought to be incomplete because it lacked 2-oxoglutarate dehydrogenase, which generates succinyl-CoA from 2-oxoglutarate. Biochemical analysis based on *Synechococcus* sp. PCC 7002, however, has demonstrated that marine cyanobacteria possess the enzymes 2-oxoglutarate decarboxylase and succinic semialdehyde dehydrogenase, which generate succinate from 2-oxoglutarate by two enzymatic reactions^34^. In *Synechocystis* 6803, additional enzymatic reactions involving the γ-aminobutyric acid (GABA) shunt generate succinate from 2-oxoglutarate^35^. In this shunt, 2-oxoglutarate is converted to glutamate, followed by conversion to GABA^35^ which is then converted to succinyl-semialdehyde, and finally to succinate^35^. In addition to these bypasses, succinate is also produced in the reductive TCA cycle in *Synechocystis* 6803 under dark, anaerobic conditions^36^. Phosphoenolpyruvate carboxylase (PEPC), which generates oxaloacetate from phosphoenolpyruvate, is a rate-limiting enzyme for succinate production under these conditions^36^. Generally, PEPCs in bacteria and plants are inhibited by aspartate and malate; however, PEPC in *Synechocystis* 6803 is uniquely insensitive to these metabolites^37^. Substitution of the glutamate residue with lysine at position 954 restores these inhibitory effects, similar to that seen for PEPC in a nitrogen-fixing cyanobacterium^37^. These recent studies demonstrate that TCA cycle enzymes in cyanobacteria possess unique properties, making biochemical characterisation of these enzymes not only intriguing, but also indispensable for understanding their metabolism.

In the present study, biochemical analysis of fumarase C from *Synechocystis* 6803 (*Sy*FumC) revealed that the reversible reactions are regulated by TCA cycle metabolites and further showed the importance of the alanine residue at position 314 for substrate affinity.

## Results

### Affinity purification and biochemical characterisation of *Sy*FumC

**Figure 1:**
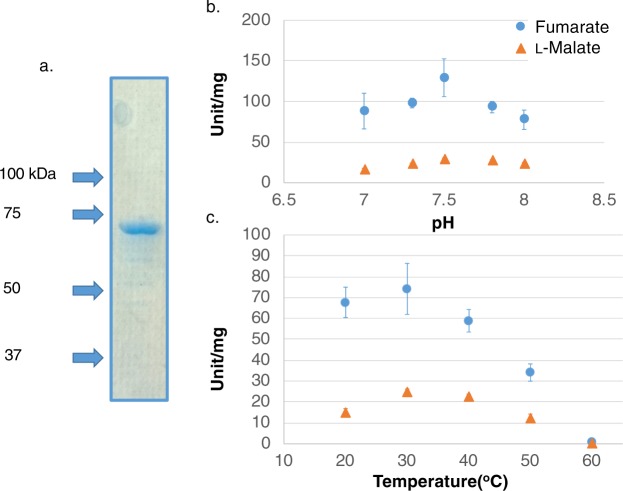
Determination of optimum conditions for *Synechocystis* sp. PCC 6803 fumarase C (*Sy*FumC). (**a**) GST-tagged *Sy*FumC after purification. Purified proteins were electrophoresed on 8% SDS-PAGE gel and stained using InstantBlue reagent. (**b**) Effect of pH on *Sy*FumC activity. Data represent the means ± SD obtained from three independent experiments. For the enzyme assay, 15 pmol of *Sy*FumC was used. One unit of *Sy*FumC activity was defined as the consumption of 1 µmol fumarate or l-malate per min. Light blue circles and orange triangles represent activity specific for fumarate and l-malate, respectively. (**c**) Effect of temperature on *Sy*FumC activity. Data represent the means ± SD obtained from three independent experiments. For the enzyme assay, 15 pmol of *Sy*FumC was used. One unit of *Sy*FumC activity was defined as the consumption of 1 µmol fumarate or l-malate per min. Light blue circles and orange triangles represent activity specific for fumarate and l-malate, respectively.

Genome sequence analysis indicated that *Synechocystis* 6803 does not possess a Class I fumarase but does express a Class II fumarase, *Sy*FumC. To evaluate the biochemical properties of *Sy*FumC, glutathione-*S*-transferase-tagged *Sy*FumC (GST-*Sy*FumC) was expressed in *E. coli* cells and purified from the soluble fraction by affinity chromatography (Figs 1a and S1). The enzymatic activity of *Sy*FumC using fumarate as a substrate (hereafter referred to as “the enzymatic activity towards fumarate”) was highest at pH 7.5 (Fig. 1b); with l-malate as a substrate (hereafter referred to as “the enzymatic activity towards l-malate”), the enzymatic activity of *Sy*FumC was also highest at pH 7.5, but was less affected by variation in pH. Enzymatic activities towards both fumarate and l-malate were highest at 30 °C and were inhibited at 60 °C (Fig. 1c). *Sy*FumC activity towards l-malate was less sensitive to temperature variation than that toward fumarate (Fig. 1c). Therefore, for the ensuing experiments, *Sy*FumC enzymatic assays were performed at pH 7.5 and 30 °C.

**Figure 2:**
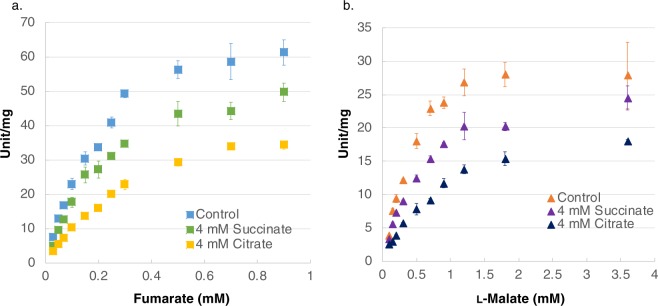
*In vitro* enzyme assays for *Sy*FumC using fumarate and l-malate as substrates. (**a**) Enzyme activity was measured by varying the fumarate concentration. The data represent the means ± SD obtained from three independent experiments. Light blue squares represent activity specific for fumarate. Yellow and green squares represent activity specific for fumarate  with 4 mM citrate and 4 mM succinate, respectively. (**b**) Enzyme activity was measured by varying the l-malate concentration. The data represent the means ± SD obtained from three independent experiments. Orange triangles represent activity specific for l-malate. Navy and purple triangles represent activity specific for l-malate with 4 mM citrate and 4 mM succinate, respectively.

Substrate saturation curves were obtained using different concentrations of fumarate (Fig. 2a) and l-malate (Fig. 2b) as substrates. The *K*_m_ of *Sy*FumC for fumarate and l-malate was 0.244 ± 0.026 and 0.478 ± 0.112 mM, respectively; the *k*_cat_ of *Sy*FumC for fumarate and l-malate was 101.2 ± 6.7 and 42.3 ± 4.3 s^−1^, respectively; and the *k*_cat_/*K*_m_ of *Sy*FumC for fumarate and l-malate was 415.1 ± 17.0 and 90.3 ± 11.3 s^−1^ mM^−1^, respectively (Table 1).

**Table 1:** Kinetic parameters of *Sy*FumC using fumarate and l-malate as substrates at 30 °C and pH 7.5.

Enzyme	Effector	*K*_m_ (mM)	*k*_cat_ (s^−1^)	*k*_cat_/*K*_m_ (s^−1^ mM^−1^)
**Using fumarate as a substrate**				
*Sy*FumC	None	0.244 ± 0.026	101.2 ± 6.7	415.1 ± 17.0
*Sy*FumC	4 mM Suc	0.256 ± 0.017	79.5 ± 2.2	311.5 ± 12.4
*Sy*FumC	4 mM Cit	0.409 ± 0.034	65.4 ± 2.1	160.2 ± 8.8
*Sy*FumC_A314E	None	0.179 ± 0.036	37.8 ± 3.0	214.8 ± 27.9
*Sy*FumC_A314E	4 mM Suc	0.214 ± 0.025	44.0 ± 2.7	206.3 ± 10.7
*Sy*FumC_A314E	4 mM Cit	0.391 ± 0.036	47.4 ± 2.1	121.7 ± 6.0
**Using** **L** **-malate as a substrate**				
*Sy*FumC	None	0.478 ± 0.112	42.3 ± 4.3	90.3 ± 11.3
*Sy*FumC	4 mM Suc	0.616 ± 0.073	34.9 ± 1.8	57.0 ± 4.0
*Sy*FumC	4 mM Cit	0.921 ± 0.042	28.0 ± 0.4	30.4 ± 1.8
*Sy*FumC_A314E	None	0.442 ± 0.020	21.6 ± 0.1	49.0 ± 2.0
*Sy*FumC_A314E	4 mM Suc	0.329 ± 0.030	12.7 ± 0.7	38.7 ± 1.6
*Sy*FumC_A314E	4 mM Cit	0.360 ± 0.020	10.4 ± 0.04	29.0 ± 1.7

Parameters were calculated using the equations described in the Materials and Methods. Cit and Suc represent citrate and succinate, respectively. The data represent the means ± SD from three independent experiments.

### Identification of effectors altering *Sy*FumC activity

**Figure 3:**
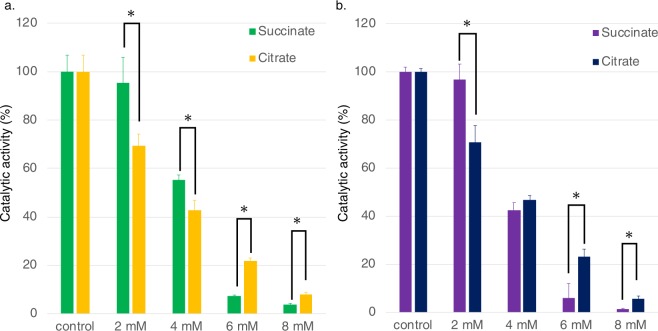
Effect of citrate and succinate on *Sy*FumC activity. Enzymatic activity was measured by varying the concentrations of citrate or succinate under conditions of substrate (fumarate and l-malate) saturation. (**a**) Yellow and green bars represent *Sy*FumC enzymatic activity towards fumarate with citrate and succinate, respectively. (**b**) Navy and purple bars represent *Sy*FumC enzymatic activity towards l-malate with citrate and succinate, respectively. Asterisk (*) indicates *P* < 0.05. The data represent the means ± SD from three independent experiments.

Effectors of *Sy*FumC activities were also evaluated. Citrate and succinate are competitive inhibitors of fumarases from pig heart, *E. coli*, and *Pisum sativum*^38–40^; therefore, the effect of citrate and succinate on *Sy*FumC was examined at various concentrations (2–8 mM) under conditions of substrate saturation (Fig. 3a,b). *Sy*FumC activity towards both fumarate and l-malate reactions decreased in a concentration-dependent manner in the presence of both citrate and succinate (Fig. 3a,b). The enzymatic activity of *Sy*FumC towards fumarate and l-malate decreased to 45–58% of control in the presence of 4 mM succinate or 4 mM citrate (Fig. 3a,b). The *K*_m_ and *k*_cat_ for fumarate and l-malate in the presence of 4 mM succinate were 0.256 ± 0.017 mM and 79.5 ± 2.2 s^−1^; and 0.409 ± 0.034 mM and 65.4 ± 2.1 s^−1^, respectively (Table 1). The *K*_m_ and *k*_cat_ for fumarate and l-malate in the presence of 4 mM citrate were 0.616 ± 0.073 mM and 34.9 ± 1.8 s^−1^; and 0.921 ± 0.042 mM and 28.0 ± 0.4 s^−1^, respectively (Table 1). The *k*_cat_/*K*_m_ for fumarate and l-malate in the presence of 4 mM succinate was 311.5 ± 12.4 and 57.0 ± 4.0, respectively; and in the presence of 4 mM citrate, it was 160.2 ± 8.8 and 30.4 ± 1.8 s^−1^ mM^−1^, respectively (Table 1).

**Figure 4:**
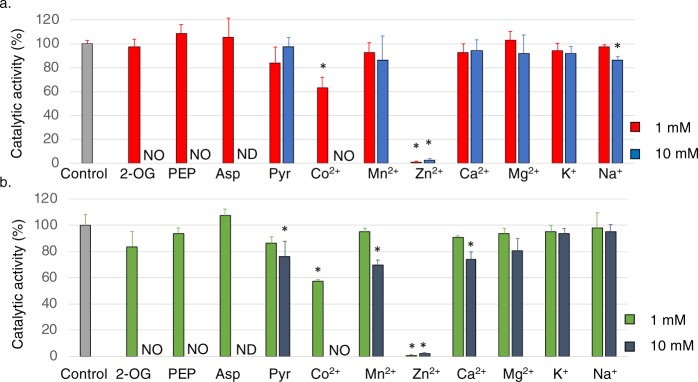
Effect of various metal ions and metabolites on *Sy*FumC activity using fumarate and l-malate as substrates. Enzymatic activity was measured with various metabolites or metal ions under conditions of substrate (fumarate and l-malate) saturation. (**a**) The grey bars represent the control data. Red and blue bars represent concentrations of 1 mM and 10 mM metabolites or metal ions, respectively, using fumarate as a substrate. (**b**) The grey bar represents the control data. Green and dark blue bars represent, respectively, using l-malate as a substrate. NO, no enzymatic activity was detected; ND, enzymatic activity not determined in this study. The data represent the means ± SD from three independent experiments. Asterisk (*) indicates *P* < 0.05. Abbrevations; 2-OG, 2-oxoglutarate; PEP, phosphoenolpyruvate; Asp, aspartate; Pyr, pyruvate.

Additional effectors of *Sy*FumC enzymatic activity were examined under conditions of substrate saturation. We selected 11 effectors which regulate the activities of enzymes in the TCA cycle and pyruvate metabolism in this cyanobacterium^37,41^. Among the 11 effectors tested, Co^2+^ and Zn^2+^ decreased *Sy*FumC activity towards fumarate and l-malate (Fig. 4a,b). At 1 mM, Co^2+^ decreased *Sy*FumC enzymatic activity towards fumarate and l-malate to 66.5% and 57.4% of control, respectively (Fig. 4a,b). The presence of 1 mM and 10 mM Zn^2+^ decreased *Sy*FumC enzymatic activity towards fumarate to 0.99% and 1.99% of control, respectively (Fig. 4a,b); similarly, 1 mM and 10 mM Zn^2+^ decreased *Sy*FumC enzymatic activity towards l-malate to 0.94% and 1.89% of control (Fig. 4a,b). At 10 mM, pyruvate and Mn^2+^ both decreased *Sy*FumC enzymatic activity towards l-malate (Fig. 4a,b). *Sy*FumC activity could not be measured in the presence of 10 mM Co^2+^, 2-oxoglutarate, and phosphoenolpyruvate due to interference of absorption by solvents (Fig. 4a,b).

### Amino acid substitution and substrate specificity of *Sy*FumC

**Figure 5:**
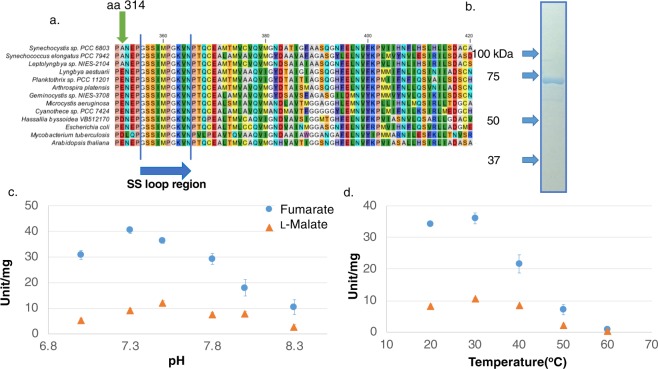
Multiple amino acid sequence alignment of Class II fumarases and determination of optimum conditions for *Synechocystis* sp. PCC 6803 fumarase C with glutamate substituted for alanine (*Sy*FumC_A314E). (**a**) The multiple alignment of 13 amino acid sequences from Class II fumarases performed by CLC Sequence Viewer. The region containing the GSSxxPxKxN sequence, called an SS loop (indicated by a blue arrow), is shown. The green arrow indicates the alanine at position 314 in *Synechocystis* sp. PCC 6803. (**b**) GST-tagged *Sy*FumC_A314E after purification. Purified proteins were electrophoresed on 8% SDS-PAGE gel and stained using InstantBlue reagent. (**c**) Effect of pH on *Sy*FumC_A314E activity. Data represent the means ± SD obtained from three independent experiments. For the enzyme assay, 15 pmol of *Sy*FumC_A314E was used. One unit of *Sy*FumC activity was defined as the consumption of 1 µmol fumarate or l-malate per min. Light blue circles and orange triangles represent activity specific for fumarate and l-malate, respectively. (**d**) Effect of temperature on *Sy*FumC_A314E. For the enzyme assay, 15 pmol of *Sy*FumC_A314E was used. One unit of *Sy*FumC activity was defined as the consumption of 1 µmol fumarate or l-malate per min. Light blue circles and orange triangles represent activity specific for fumarate and l-malate, respectively.

Amino acid sequences were compared by multiple sequence alignment analysis, using amino acid sequences of fumarases from other cyanobacteria, *E. coli*, *Mycobacterium tuberculosis*, and *Arabidopsis thaliana* (Fig. 5a). The amino acid residue at position 314 of *Sy*FumC was found to be alanine; however, except for *Synechococcus elongatus* and *Leptolyngbya* sp., the equivalent residues of other fumarases are either glutamate or aspartate (Fig. 5a).

The impact of the alanine residue at position 314 of *Sy*FumC was examined by substituting glutamate for alanine at position 314, and the recombinant protein was named *Sy*FumC_A314E. The *Sy*FumC_A314E protein was expressed in *E. coli* and purified by affinity chromatography (Figs 5b and S2). The optimal pH for *Sy*FumC_A314E enzymatic activity towards fumarate was pH 7.3 and the optimal temperature was 30 °C (Fig. 5c). *Sy*FumC_A314E was inactivated at 60 °C (Fig. 5d). Glutamate substitution had less impact at optimal pH and temperature using l-malate as a substrate (Fig. 5c,d). The maximum enzymatic activity of *Sy*FumC_A314E for fumarate at the optimal pH and temperature decreased to 28.1% and 48.6% of *Sy*FumC activity, respectively (Fig. 5c,d); the maximum enzymatic activity of *Sy*FumC_A314E for l-malate at the optimal pH and temperature decreased to 41.5% and 41.6% of *Sy*FumC activity, respectively (Fig. 5c,d).

**Figure 6:**
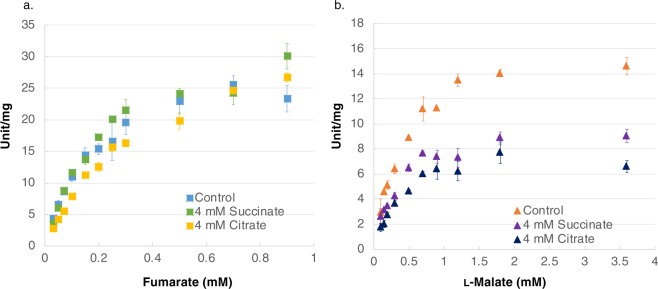
*Sy*FumC_A314E enzyme assays using fumarate and l-malate as substrates. (**a**) Enzyme activity was measured by varying the fumarate concentration. The data represent the means ± SD obtained from three independent experiments. Light blue squares represent activity specific for fumarate. Yellow squares and green squares represent activity specific for fumarate with 4 mM citrate and with 4 mM succinate, respectively. (**b**) Enzyme activity was measured by varying the l-malate concentration. The data represent the means ± SD obtained from three independent experiments. Orange triangles represent activity specific for l-malate. Navy triangles and purple triangles represent activity specific for l-malate with 4 mM citrate and 4 mM succinate, respectively.

The enzymatic activity of *Sy*FumC_A314E was measured with various substrate concentrations to obtain saturation curves and calculate the kinetic parameters (Fig. 6a,b). The *K*_m_ values for *Sy*FumC_A314E towards fumarate and l-malate were 0.179 ± 0.036 and 0.442 ± 0.020 mM, respectively (Table 1), showing reductions to 73.4% and 92.5% of *Sy*FumC values for fumarate and l-malate, respectively (Table 1). The *k*_cat_ values for *Sy*FumC_A314E towards fumarate and l-malate were 37.8 ± 3.0 and 21.6 ± 0.1 s^−1^, respectively (Table 1).

The *K*_m_ and *k*_cat_ for *Sy*FumC_A314E were examined in the presence of 4 mM succinate or 4 mM citrate. The *K*_m_ for *Sy*FumC_A314E using fumarate and l-malate as substrates in the presence of 4 mM succinate was 0.214 ± 0.025 and 0.329 ± 0.030 mM, respectively (Table 1). The *K*_m_ for *Sy*FumC_A314E in the presence of 4 mM citrate, using fumarate and l-malate as substrates, was 0.391 ± 0.036 and 0.360 ± 0.020 mM, respectively (Table 1). The *k*_cat_ values for *Sy*FumC_A314E with fumarate and l-malate as substrates, in the presence of 4 mM succinate, were 44.0 ± 2.7 and 12.7 ± 0.7 s^−1^, respectively (Table 1). The *k*_cat_ values for *Sy*FumC_A314E with fumarate and l-malate as substrates, in the presence of 4 mM citrate, were 28.0 ± 0.4 and 10.4 ± 0.04 s^−1^, respectively (Table 1). The *k*_cat_/*K*_m_ ratios for *Sy*FumC_A314E with fumarate and l-malate as substrates were 206.3 ± 10.7 and 38.7 ± 1.6, respectively, in the presence of 4 mM succinate; and 121.7 ± 6.0 and 29.0 ± 1.7 s^−1^ mM^−1^, respectively, in the presence of 4 mM citrate (Table 1).

**Figure 7:**
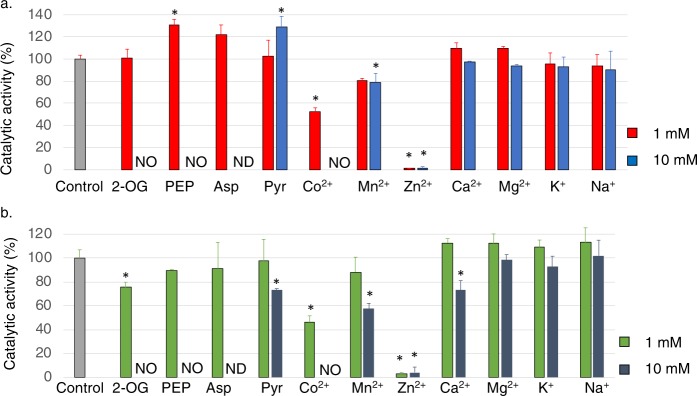
Effect of various metal ions and metabolites on *Sy*FumC_A314E activity towards fumarate and l-malate. Enzymatic activity was measured with various metabolites or metal ions under conditions of substrate (fumarate and l-malate) saturation. (**a**) The grey bar represents the control data. Red and blue bars represent concentrations of 1 mM and 10 mM metabolites or metal ions, respectively, using fumarate as a substrate. (**b**) The grey bar represents the control data. Green and dark blue bars represent concentrations of 1 mM and 10 mM metabolites or metal ions, respectively, using l-malate as a substrate. NO, no enzymatic activity was detected; ND, enzymatic activity not determined in this study. The data represent the means ± SD from three independent experiments. Asterisk (*) indicates *P* < 0.05.

Finally, 11 effectors (metabolites and metal ions) were tested for their impact on *Sy*FumC_A314E activity. At 1 mM, Zn^2+^ eliminated *Sy*FumC_A314E enzymatic activity towards fumarate and l-malate (Fig. 7a,b). Also, at 1 mM, Co^2+^ decreased *Sy*FumC_A314E enzymatic activity towards fumarate and l-malate to 54.6% and 47.6% of control, respectively (Fig. 7a,b). *Sy*FumC_A314E enzymatic activity towards fumarate and l-malate was abolished with 10 mM Mn^2+^ (Fig. 7a,b). *Sy*FumC_A314E enzymatic activity towards fumarate was marginally upregulated in the presence of 1 mM phosphoenolpyruvate (Fig. 7a); and at 10 mM, pyruvate promoted *Sy*FumC_A314E enzymatic activity towards fumarate while enzymatic activity towards l-malate was suppressed (Fig. 7a,b).

## Discussion

In this study, we performed the first biochemical analysis of class II fumarase in cyanobacteria, revealing that the modification of a residue at position 314 relieved the inhibition by citrate and succinate and reduced *k*_cat_ of *Sy*FumC. Previous studies have shown that the optimal pHs for fumarases from the thermophilic archaebacterium *Sulfolobus solfataricus* and from marine microorganisms are 8.0 and 8.5, respectively^42,43^. *Sy*FumC is more active at a lower pH than these fumarases (Fig. 1b). The optimal pHs for fumarases from the eukaryotes *Saccharomyces cerevisiae* and *Rhizopus oryzae* are pH 7.5 and 7.2^44,45^, respectively, and are thus similar to that for *Sy*FumC (Fig. 1b). The growth pH ranges for *S. solfataricus* and *S. cerevisiae* are pH 1.0–5.8 and pH 4.0–4.5, respectively^46,47^, values inconsistent with the optimum pH for fumarases. *Synechocystis* 6803 shows optimal biomass production at pH 7.5^48^, a pH for growth consistent with optimal enzymatic activity.

The optimal temperature for *Sy*FumC activity was 30 °C, which is similar to that of fumarases from *R. oryzae* (30 °C) and the mesophilic *Streptomyces coelicolor* (30 °C), but lower than for bacterial and archaeal fumarases in *Streptomyces lividans* (45 °C), thermophilic *Streptomyces thermovulgaris* (50 °C) and *Thermus thermophilus* (85 °C), *S. solfataricus* (85 °C), and marine microorganisms (55 °C)^42,43,45,49–51^. *S. thermovulgaris*, *T. thermophilus*, and *S. solfataricus* are thermophilic organisms that thrive in hot springs, and higher optimal temperatures are not unexpected. The optimal temperature for *Synechocystis* 6803 growth is 30–32 °C^52^, which is close to the optimal temperature for *Sy*FumC activity. The optimal temperatures for two other *Synechocystis* enzymes, *Sy*PEPC and d-lactate dehydrogenase, are 30 °C and 30–40 °C, respectively^37,53^. The optimal temperature for *Sy*FumC enzymatic activity is thus similar to that of other *Synechocystis* enzymes involved in primary carbon metabolism.

The *K*_m_ for *Sy*FumC enzymatic activity towards l-malate was higher than that for fumarate (Table 1). Fumarases from other organisms like *E. coli*^54^, *Corynebacterium glutamicum*^55^, *S. solfataricus*^42^, and marine microorganisms^43^ are similar; the *K*_m_ ranges for fumarate and l-malate are 0.12–0.48 and 0.30–3.15 mM, respectively. Intracellular metabolite analysis using ^13^C-labeled glucose *Synechocystis* 6803 demonstrated that the number of ^13^C-atoms in malate was higher than in fumarate by approximately 5-fold at 37 °C in *Synechocystis* 6803^56^. This ^13^C flux analysis indicates that the conversion reaction from fumarate to l-malate is stronger than that from l-malate to fumarate under aerobic conditions. Previous reports showed that the absolute concentration of l-malate (µmol/g dry cell weight) is higher than that of fumarate in *Synechocystis* 6803^57^, indicating that *Sy*FumC preferentially catalyses the reaction generating l-malate from fumarate. A different study shows that intracellular fumarate and malate pool sizes peak at 24 h under dark, anaerobic conditions, and then decrease during cultivation under the same conditions^36^. Carbon flow in the TCA cycle changes from using fumarate to using l-malate during dark, anaerobic incubation^36^. Nevertheless, intracellular l-malate concentrations (µmol/g dry cell weight) are still higher than fumarate concentrations^36^. The substrate specificity of *Sy*FumC for fumarate well explains these results (Table 1). The *K*_m_ of a malate dehydrogenase from *Synechocystis* 6803 (*Sy*MDH) that catalyses the reversible conversion of l-malate and oxaloacetate is approximately 210-fold higher for the oxidative than for the reductive reaction and the catalytic efficiency of *Sy*MDH is higher for the reductive than for the oxidation reaction^58^. Thus, both *Sy*MDH and *Sy*FumC preferentially catalyse reactions that produce l-malate. A previous study suggested that organic acids in the TCA cycle, including l-malate, play critical roles in the storage of carbon sources under nitrogen starvation^31^. *Sy*FumC and *Sy*MDH may preferentially convert metabolites to l-malate to promote this process.

The *k*_cat_ for *Sy*FumC enzymatic activity towards l-malate was lower than those for fumarase C from *E. coli*^54^ and Class II fumarases from marine microorganisms and *T. thermophilus*^43,51^, and *Homo sapiens*^59^ (Table 2). Fluxome analysis indicates that TCA cycle fluxes in *Synechocystis* 6803 remain constant under dark, photoheterotrophic, photomixotrophic, and photoautotrophic conditions, suggesting that the cyanobacterial TCA cycle does not naturally produce energy sources^33^. Metabolic reaction catalysed by a fumarase is inclined to the flux from fumarate to malate in all condition^33^. In addition, molar-based, widely targeted metabolic profiling analysis reveals that concentrations of TCA cycle metabolites are lower than those of glycolysis metabolites^57^. We compared fumarases of organisms in different kingdoms (Table 2). The *k*_cat_ of *Sy*FumC is lower than *k*_cat_ of other microorganisms fumarases. *K*_m_ of *Sy*FumC using fumarate as a substrate is lower than *K*_m_ of other microorganisms fumarases using fumarate as a substrate except *K*_m_ for fumarate of *E. coli* (Table 2). Since the specific activity of *Sy*FumC is lower than those of other organisms, the turnover number limits *Sy*FumC activity *in vitro* (Table 2). These results were consistent with a less active TCA cycle in cyanobacteria^33^. On the other hand, *k*_cat_/*K*_m_ of *Sy*FumC using l-malate was higher than its of *C. glutamicum* fumarase (Table 2). The result indicates the reductive TCA cycle is relatively efficient compared to other organisms, consistent with a previous study^58^.

**Table 2:** Kinetic parameters of other class II fumarases.

Substrate	Fumarate			l-malate		
Organisms	*K*_m_ (mM)	*k*_cat_ (s^−1^)	*k*_cat_/*K*_m_ (s^−1^ mM^−1^)	*K*_m_ (mM)	*k*_cat_ (s^−1^)	*k*_cat_/*K*_m_ (s^−1^ mM^-1^)
*Synechocystis* sp. PCC 6803 (This study)	0.24	1.0 × 10^2^	4.1 × 10^2^	0.48	4.2 × 10	9.0 × 10
*Corynebacterium glutamicum* (pH 7.0/TES-NaOH)^50^	0.38	4.30 × 10^2^	1.1 × 10^3^	1.80	1.1 × 10^2^	6.1 × 10
*Escherichia coli* (Native)^49^	0.21	1.1 × 10^3^	5.6 × 10^3^	0.86	5.9 × 10^2^	6.9 × 10^2^
*Homo sapiens* ^58^	NR	NR	NR	1.90	1.5 × 10^2^	8.0 × 10
Marine sample (FumF)^38^	0.48	9.1 × 10^2^	1.9 × 10^3^	3.20	3.2 × 10^2^	1.0 × 10^2^
*Streptomyces lividans* (pH 7.3)^45^	NR	NR	NR	15.0	2.7 × 10^2^	1.8 × 10
*Thermus thermophilus* ^46^	NR	NR	NR	1.00	1.0 × 10^3^	1.0 × 10^3^

Citrate is known as a competitive inhibitor of fumarases from pig heart^38^ and *E. coli*^39^, and citrate also inhibited *Sy*FumC activity (Fig. 3, Table 1). Mitochondrial fumarase of a higher plant, *P. sativum*, is inhibited by 53% in the presence of 20 mM citrate, using l-malate as a substrate^60^. The activities of *A. thaliana* mitochondrial and cytosolic fumarases decrease to 15–37% in the presence of citrate^61^. Succinate is also known as an inhibitor of fumarases of pig and *P. sativum*^60,62^, and it also inhibited *Sy*FumC (Fig. 3). Succinate at 100 mM inhibits *P. sativum* fumarase; the activity decreases to 55% using l-malate as a substrate^60^. The sensitivity of *Sy*FumC to citrate and succinate was nearly identical with either fumarate and l-malate as substrates (Fig. 3a,b). Absolute quantification of metabolites (µmol/g dry cell weight) in *Synechocystis* 6803 shows that intercellular concentrations of citrate, succinate, fumarate, and malate were 2.16, 0.323, 0.163, and 0.182, respectively^57^. These concentrations suggest that citrate and succinate are inhibitory under typical physiological conditions and the reduced *Sy*FumC activity may be one cause of the low fluxes through the TCA cycle observed in *Synechocystis* 6803.

Additionally, *Sy*FumC activity was also inhibited by several metabolites and divalent cations (Fig. 4a,b). *P. sativum* mitochondrial fumarase activity towards l-malate was decreased to 14% at 10 mM pyruvate^60^. *A. thaliana* mitochondrial fumarase is activated to 178% by pyruvate using l-malate as a substrate^61^. At 10 mM, pyruvate marginally inhibited *Sy*FumC activity towards l-malate (Fig. 4b), and the effect of pyruvate on fumarases is different among photosynthetic organisms. Zn^2+^ strongly inhibited *Sy*FumC enzymatic activity using fumarate and l-malate as substrates (Fig. 4a,b). This inhibition is greater than reported for a fumarase from a marine microorganism (FumF), FumF enzymatic activity reduces to 45% by 5 mM Zn^2+^ ^43^. In contrast, a fumarase from *R. oryzae* is not inhibited by Zn^2+^ ^45^. The addition of 10 mM Ca^2+^ was less inhibitory by other divalent cations for *Sy*FumC and *Sy*FumC_A314E activity towards l-malate (Figs 4b and 7b), but the *R. oryzae* fumarase is slightly stimulated by 9.0 mM Ca^2+^ using l-malate as a substrate^45^. Compared with other organisms fumarases, *Sy*FumC was differently inhibited by metabolites and metal ions. These results indicated that the regulation by metabolites and metals was not conserved among fumarases. Three TCA cycle enzymes (*Sy*MDH, isocitrate dehydrogenase, and citrate synthase) in *Synechocystis* 6803 are regulated by divalent cations, particularly Mg^2+^ ^41,58,63^. *Sy*FumC was severely inhibited by Zn^2+^, not by Mg^2+^, indicating the different regulatory manner of enzymatic activities in the TCA cycle in this cyanobacterium.

The alanine at position 314 is close to the sequence called the SS loop, a motif associated with substrate binding and catalytic activity^1^. The optimal pH and temperature for *Sy*FumC_A314E were similar to the optima for *Sy*FumC (Fig. 5c,d), but substrate affinity was enhanced by the A314E substitution (Fig. 6). The *K*_m_ values for *Sy*FumC_A314E activity towards fumarate and l-malate were not significantly different to those for *Sy*FumC activity towards the same substrates (Table 1). Compared to *Sy*FumC, the *k*_cat_ for *Sy*FumC_A314E activity towards fumarate and l-malate decreased to 0.37-fold and 0.51-fold, respectively (Table 1). The *k*_cat_ for an *E. coli* recombinant FumC with substituting glutamine for glutamate at position 315 near the SS loop was decreased by 10-fold when using fumarate and l-malate as substrates, but no effect was observed for *K*_m_ values^54^. This glutamate residue at position 315 in *E. coli* is correspond to a glutamate at position 316 in *Sy*FumC (Fig. 5). These results indicate that amino acids near the SS-loop determine the maximum rate of reaction of bacterial fumarases. Other group demonstrate that the alanine at position 347 in *S. coelicolor* fumarase (correspond to the glutamine at position 354 in *Sy*FumC) is important for thermostability^49^, and the improvement of the thermostability in *Sy*FumC can be future theme for cyanobacterial fumarases.

**Figure 8:**
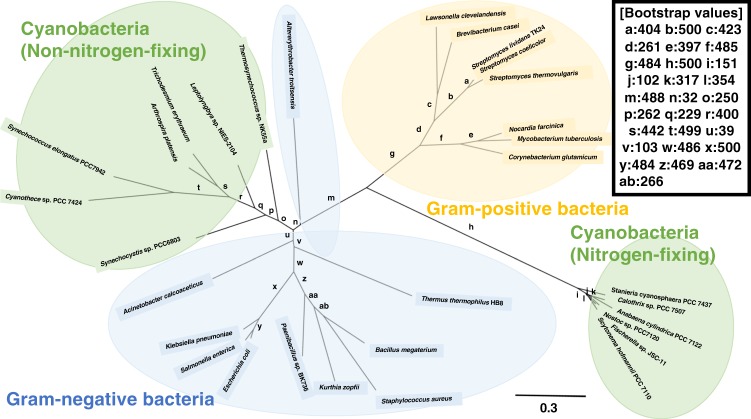
Phylogenetic analysis of bacterial class II fumarases. Amino acid sequences of 31 bacterial fumarase obtained from GenBank were aligned using CLC Sequence Viewer ver. 8.0. A maximum-likehood tree based on 431 preserved amino acid residues was designed using PhyML online (http://www.atgc-montpellier.fr/phyml/). Bootstrap values were calculated by 500 replications.

Phylogenetic analysis revealed that *Sy*FumC belongs to non-nitrogen-fixing cyanobacterial clade (except *Trichodesmium erythraeum*) being different from nitrogen-fixing cyanobacteria and Gram-negative bacteria (Fig. 8). Combined with previous data, the present results demonstrate that important amino acid residues are conserved between enteric bacteria and non-nitrogen-fixing cyanobacteria. However, succinate inhibited the *Sy*FumC activity (Fig. 2a), while fumarase in *C. glutamicum* is not inhibited by succinate^55^, indicating the sensitivity of fumarases to the effetors are different among the clades. These differences were derived from several amino acid substitutions; the inhibitory effects of citrate and succinate on *Sy*FumC activity were reduced by the alanine to glutamate amino acid substitution (Figs 2a and 6a). The effects of some metabolites were also changed after the amino acid substitution. Pyruvate at 10 mM and phosphoenolpyruvate at 1 mM activated enzymatic activity with fumarate as a substrate (Fig. 7a), and 10 mM pyruvate and 1 mM 2-oxoglutarate inhibited *Sy*FumC_A314E activity. Additionally, 10 mM pyruvate elicited different effects depending on whether fumarate or l-malate were used as substrate (Fig. 7a,b). Pyruvate and PEP are compounds upstream of fumarase in the TCA cycle, and it is considered to be activated to acquire reducing power for fumarate.

The present results show that *Sy*FumC has a higher affinity for fumarate than for l-malate. The enzymatic activity of *Sy*FumC towards fumarate and l-malate were inhibited by citrate and succinate. Current study, a biochemistry of a fumarase in a new clade, contributes to understanding the diversity of the TCA cycle in bacteria, and potentially leads to the metabolic engineering using cyanobacteria.

## Methods

### Vector construction and expression of recombinant proteins

The genomic region of *Synechocystis* 6803 including the *fumC* (slr0018) open reading frame (ORF) with the *Bam*HI-*Xho*I fragment was amplified by PCR using the KOD -plus- neo DNA polymerase (Toyobo, Osaka, Japan) and the following primer set: forward, 5′-GAAGGTCGTGGGATCATGGTAAATTCCCACCGC-3′ and reverse, 5′-GATGCGGCCGCTCGAGCTAGTCAGCAATCGGGG-3′; the *Bam*HI and *Xho*I restriction enzymes were obtained from TakaraBio (Shiga, Japan). The resultant fragment was cloned into the *Bam*HI-*Xho*I sites of pGEX5X -1 (GE Healthcare Japan, Tokyo, Japan). Amino acid substitution was performed commercially by TakaraBio (Shiga, Japan). For *Sy*FumC_A314E, the region +940–942 from the start codon in the *fumC* ORF was changed from GCC to GAA.

These vectors were transformed into *E. coli* DH5α cells (TakaraBio) and 5 L of transformed *E. coli* were cultivated in LB medium at 30 °C with shaking (150 rpm); protein expression was induced overnight in the presence of 0.01 mM isopropyl β-D-1-thiogalactopyranoside (Wako Chemicals, Osaka, Japan).

### Affinity purification of recombinant proteins

Affinity chromatography for protein purification was performed as previously described^33^. Harvested DH5α cells suspended in 40 mL PBST (1.37 M NaCl, 27 mM KCl, 81 mM Na_2_HPO_4_·12H_2_O, 14.7 mM KH_2_PO_4_, and 0.05% Tween 20) were disrupted by sonication (VC-750, EYELA, Tokyo, Japan) 10 times for 20 s at 20% intensity. The disrupted cells were removed by centrifugation at 5800 × *g* for 2 min at 4 °C. The supernatant was transferred to a 50-mL tube and placed on ice, and 560 μL of Glutathione Sepharose 4B resin (GE Healthcare Japan) was mixed into the supernatant, followed by gentle shaking for 30 min. After centrifugation (5800 × *g* for 2 min at 4 °C), the supernatant was removed and the resin was re-suspended in 700 μL of PBST. After washing five times, recombinant proteins were eluted five times with 700 μL of GST elution buffer [50 mM Tris-HCl (pH 8.0), 10 mM reduced glutathione]. Proteins were concentrated with a Vivaspin 500 MWCO 50000 device (Sartorius, Göttingen, Germany), and protein concentrations were analysed with a Pierce BCA Protein Assay Kit (Thermo Scientific, Rockford, IL). SDS-PAGE was performed to analyse protein purification with staining using InstantBlue (Expedion Protein Solutions, San Diego, CA).

### Enzyme assay for *Sy*FumC

*Sy*FumC activity was measured using 15 pmol *Sy*FumC mixed in 1 mL of an assay solution (100 mM Tris-HCl [pH 7.5] and 10 mM fumarate or 10 mM l-malate). Absorbance at 250 nm was monitored using a Shimadzu UV-1850 (Shimadzu, Kyoto, Japan). One unit of *Sy*FumC activity was defined as the consumption or the generation of 1 µmol fumarate per min. *K*_m_ and *V*_max_ were calculated by curve fitting using KaleidaGraph v4.5 software, and *k*_cat_ was calculated from the *V*_max_. Results were plotted as graphs of rate of reaction against substrate concentration.

### Statistical analysis

The *P*-values were calculated using paired two-tailed Student’s *t*-tests with Microsoft Excel for Windows (Redmond, WA, USA). All results were obtained from three or four independent experiments.

## Supplementary information

Supplementary figures
