# Supplementary material for: Biochemical characterisation of fumarase C from a unicellular cyanobacterium demonstrating its substrate affinity, altered by an amino acid substitution
Source: Sci Rep. 2019 Jul 23;9:10629. doi: 10.1038/s41598-019-47025-7 (PMC6650407; doi:10.1038/s41598-019-47025-7)
Supplement: Supplementary file 1 — Supplementary figures [file 41598_2019_47025_MOESM1_ESM.pdf]

Biochemical characterisation of fumarase C from a unicellular cyanobacterium demonstrating its substrate affinity, altered by an amino acid substitution

Noriaki Katayama, Masahiro Takeya, Takashi Osanai*

School of Agriculture, Meiji University, 1-1-1, Higashimita, Tama-ku, Kawasaki, Kanagawa 214-8571, Japan

*Corresponding author: Dr. Takashi Osanai, Meiji University. 1-1-1 Higashimita, Tama-ku, Kawasaki, Kanagawa 214-8571, Japan

Tel: +81-44-934-7103; Fax: +81-44-934-7103; e-mail: tosanai@meiji.ac.jp

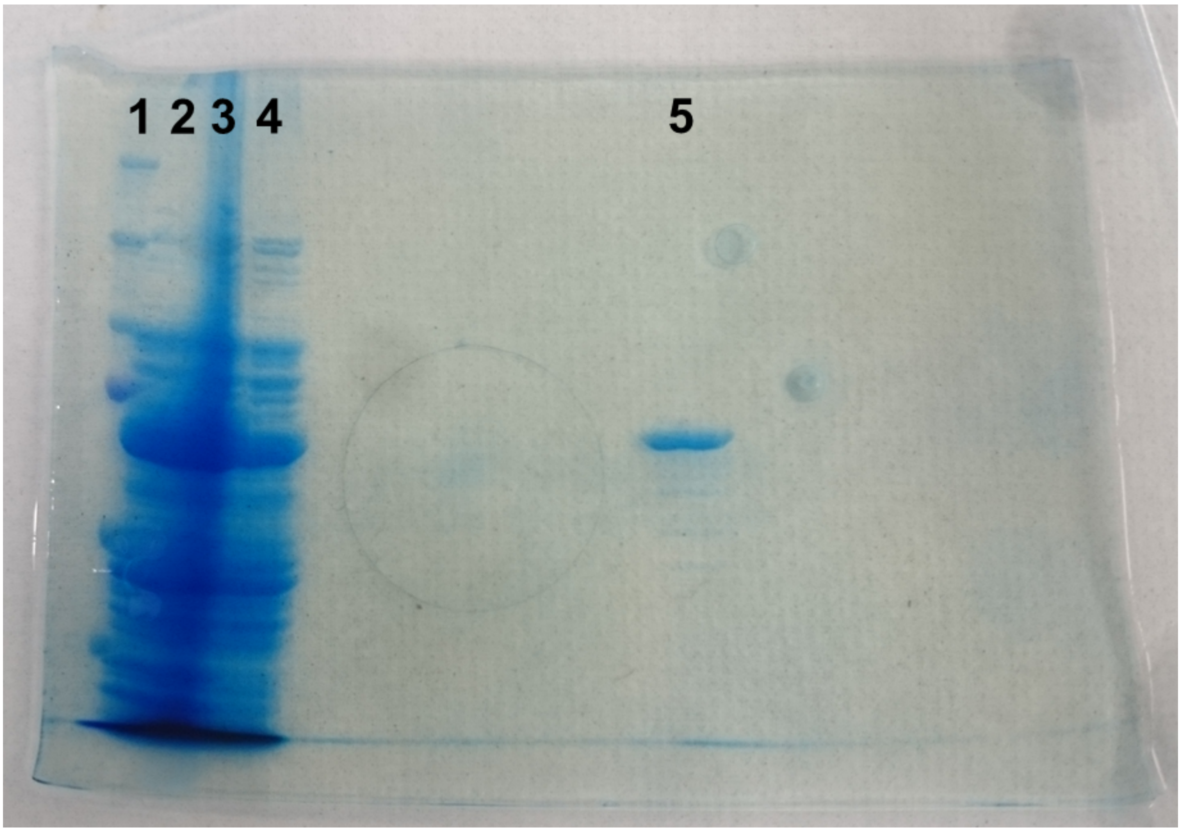

Figure S1. **GST-tagged *Sy*FumC after purification.** Fractions from the purification steps were electrophoresed on 8% SDS-PAGE gel and stained using InstantBlue reagent. (1) Molecular weight marker, (2) crude extract, (3) flow through, (4) wash fraction, (5) purified fraction. Lane (5) was in Fig.1a.

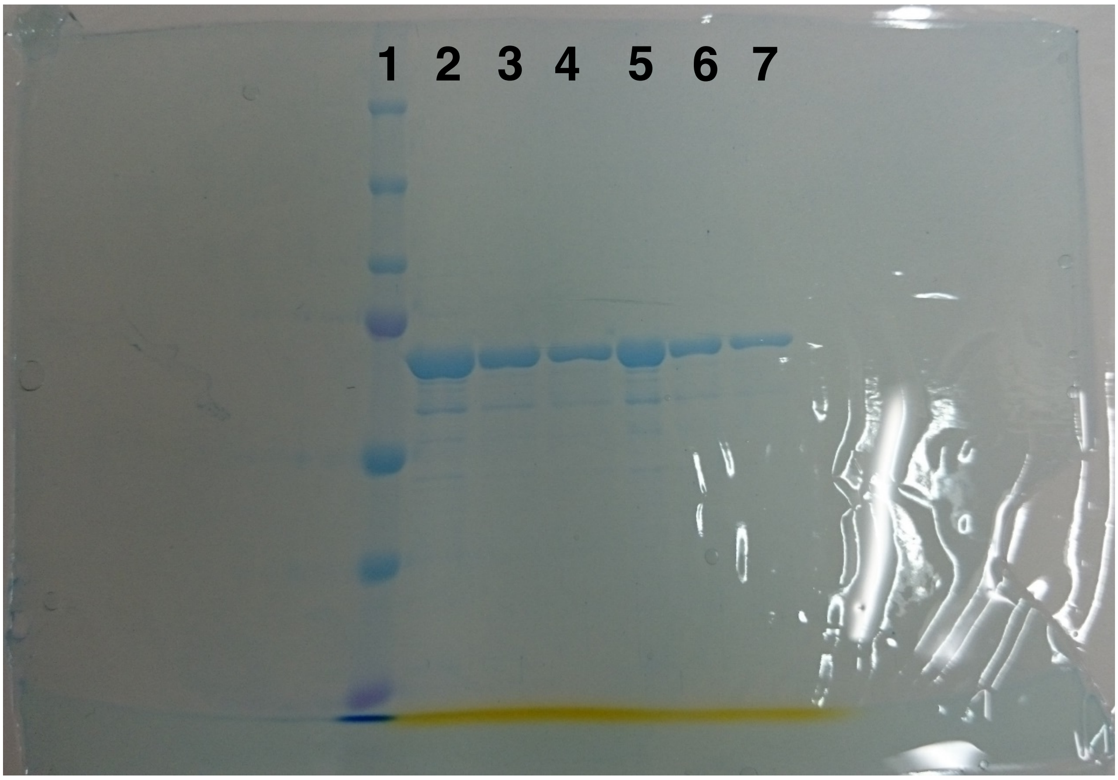

Figure S2. GST-tagged *Sy*FumC_A314E after purification. The fractions of purified proteins were electrophoresed on 8% SDS-PAGE gel and stained using InstantBlue reagent. (1) Molecular weight marker, (2, 5) purified *Sy*FumC_A314E proteins with 50% concentration, (3, 6) purified *Sy*FumC_A314E proteins with 25% concentration, (4, 7) purified *Sy*FumC_A314E proteins with 12.5% concentration. Lane (4) was used in Fig.5a.
